# Exploiting Multi-Level Parallelism for Stitching Very Large Microscopy Images

**DOI:** 10.3389/fninf.2019.00041

**Published:** 2019-06-04

**Authors:** Alessandro Bria, Massimo Bernaschi, Massimiliano Guarrasi, Giulio Iannello

**Affiliations:** ^1^Department of Electrical and Information Engineering, University of Cassino and Southern Latium, Cassino, Italy; ^2^Consiglio Nazionale delle Ricerche—CNR, Rome, Italy; ^3^CINECA Interuniversity Consortium, Casalecchio di Reno, Italy; ^4^Department of Engineering, University Campus Bio-Medico, Rome, Italy

**Keywords:** 3D microscopy, stitching, terabyte images, parallel processing, data partitioning, GPU

## Abstract

Due to the limited field of view of the microscopes, acquisitions of macroscopic specimens require many parallel image stacks to cover the whole volume of interest. Overlapping regions are introduced among stacks in order to make it possible automatic alignment by means of a 3D stitching tool. Since state-of-the-art microscopes coupled with chemical clearing procedures can generate 3D images whose size exceeds the Terabyte, parallelization is required to keep stitching time within acceptable limits. In the present paper we discuss how multi-level parallelization reduces the execution times of TeraStitcher, a tool designed to deal with very large images. Two algorithms performing dataset partition for efficient parallelization in a transparent way are presented together with experimental results proving the effectiveness of the approach that achieves a speedup close to 300×, when both coarse- and fine-grained parallelism are exploited. Multi-level parallelization of TeraStitcher led to a significant reduction of processing times with no changes in the user interface, and with no additional effort required for the maintenance of code.

## 1. Introduction

State-of-the-artmicroscopes (Dodt and et al., 2007; Silvestri et al., 2012), coupled with chemical clearing procedures to render brain tissue transparent (Chung et al., 2013) can generate 3D images having size in the order of Terabyte (TB) at high throughput. Processing and manipulation of these images require new software tools to perform a number of functions from stitching to visualization, to analysis.

Due to the limited field of view of the microscopes, acquisitions of macroscopic specimens (e.g., whole mammalian brains) require many parallel image stacks (also referred to as *tiles* in the following) to cover the whole volume of interest. Hence, multiple tiles, each composed by thousands of slices, are acquired using motorized stages. For volumes of approximately 1 cm^3^ at sub-micrometer resolution, the size of acquired data may easily exceed the Teravoxel. Since tile positions provided by the stages are not sufficient to determine a reliable displacement between tiles, an overlapping region is introduced with the purpose of making possible the automatic combination of the tiles by means of a stitching software tool.

A 3D stitching software capable to deal with these huge volumetric images was our first effort, and in 2012 we released the TeraStitcher (Bria and Iannello, 2012, 2015). This multi-platform tool, running under Linux, Mac OS, and Windows, has been adopted by many groups working in the field and it has been successfully used on images of 1 TB and more. However, that first version of TeraStitcher had some limitations, so we decided to enhance it so as to make it capable of dealing more effectively with over Terabyte-sized images.

Here we report our experience in parallelizing those parts of TeraStitcher that dominate the overall stitching time. We adopted a multi-process parallelization strategy based on launching multiple instances of TeraStitcher that process concurrently different portions of the input dataset. This approach has the following advantages: (i) it avoids the need of maintaining multiple versions of the code; (ii) it does not require any interprocess communication; and (iii) it is suitable for distributed computing on a computer cluster. This strategy requires a nontrivial partitioning of the dataset to minimize the overhead due to additional I/O operations. A contribution of the paper therefore consists of two algorithms performing the partitioning in an effective way, and thanks to the parallelization approach adopted, these algorithms are kept separate from the stitching logic. Additionally, to further speedup the execution of the most critical part of the computation, we have implemented it in CUDA to exploit fine-grained parallelism supported by Graphic Processing Units (GPUs).

Extensive experimental results, together with a detailed performance analysis, confirm that the approach allows a reduction of stitching time by at least one order of magnitude. Moreover, they point out that both coarse- and fine-grained parallelism may co-exist and lead to a reduction of stitching time that exceeds two orders of magnitude.

The rest of the paper is organized as follows. In section 2 we summarize related work and the main features of TeraStitcher that motivate parallelization. In section 3 we describe the overall parallelization strategy adopted. In sections 4 and 5 the partition algorithms and some implementation details regarding the parts of the tool running in parallel are discussed. In section 6 performance results are presented, while section 7 concludes the paper.

## 2. Related Work and Background

Several tools have been developed in the last ten years for stitching microscopy images, however most of them are not adequate to stitch 3D Teravoxel-sized datasets because they were designed under different assumptions (Emmenlauer et al., 2009; Preibisch et al., 2009; Yu and Peng, 2011; Chalfoun et al., 2017). For example, MIST (Chalfoun et al., 2017) has been recently proposed as a tool for rapid and accurate stitching of large 2D time-lapse mosaics. Although it has been designed to deal with very large datasets and it exploits different sources of parallelism to improve stitching performance, the tool can handle only 2D images each with a typical size of a few Gigabytes.

Recently, Imaris has announced a standalone commercial application capable to precisely aligning and fusing 2D, 3D, or 4D Terabyte-sized images (Bitplane, 2018). Although it is very likely that their tool uses at least multi-threading to efficiently exploit modern multi-core architectures, no information about its real capabilities and performance is available. To the best of our knowledge, the only noncommercial tool designed to handle Terabyte-sized 3D images is BigStitcher (Hörl et al., 2018), an evolution recently released of the tool described in Preibisch et al. (2009) and distributed as a plugin of Fiji (Schindelin et al., 2012). BigStitcher provides several functionalities besides stitching. It handles and reconstructs large multi-tile, multi-view acquisitions compensating all major optical effects. It also uses parallelism at thread level to speedup the stitching process.

As already stated TeraStitcher is able to stitch very large images (Bria and Iannello, 2012). It performs stitching in six steps: (i) import of the unstitched dataset; (ii) pairwise tiles displacement computation; (iii) displacement projection; (iv) displacement thresholding; (v) optimal tiles placement; and (vi) tiles merging and final multiresolution image generation. To improve flexibility, steps (i–v) generate an xml file representing, in a compact and structured form, the input of the next step. This enables running the steps separately, manually intervening to correct errors of single steps, and changing the implementation of one step without affecting the others. While the interested reader may find in Bria and Iannello (2012) a detailed description of each step, here we focus only on implementation issues related with parallelization of displacement computation and tile merging that are, by far, the most time consuming steps in the stitching pipeline and motivated our parallelization work.

**Figure 1:**
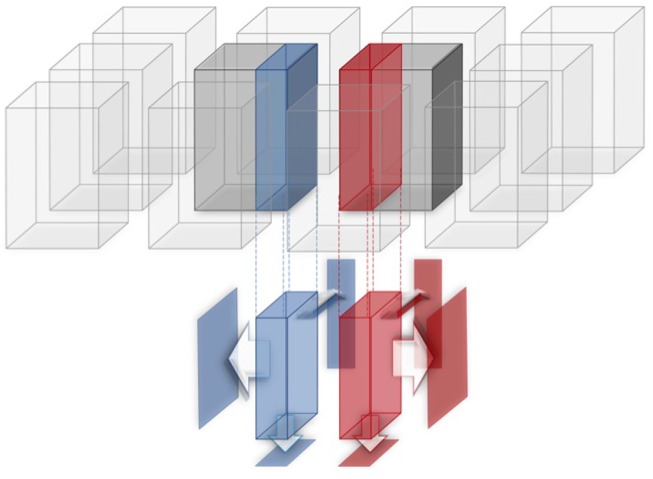
An NCC map is computed for homologous projections (MIPs) of the two overlapping (blue and red) regions of adjacent tiles.

Pairwise tiles displacement computation (*alignment step* in the following) aims at correcting the small alignment errors between adjacent tiles introduced by the microscope motorized stages. To correct the alignments, TeraStitcher uses an algorithm based on a Maximum Intensity Projection (MIP) of the overlapped area between any two adjacent tiles, and on the search of a maximum of the Normalized Cross Correlation (NCC) among pairs of homologous projections from both tiles (see Figure 1). Finding the maximum of NCC is by far the most computationally intensive part of the pairwise tiles displacement computation. It requires to move one of the two MIPs with respect to the other in any direction in order to compute a map of NCCs. The number of floating-point operations needed to compute that map depends on the size of the MIPs and of the computed NCC map. The workload associated to the pairwise tiles displacement computation is therefore an increasing function of: (i) the number of adjacent tiles; (ii) the size of the overlapping region between adjacent tiles; (iii) the size of the NCC map to be computed; (iv) the number of sub-stacks in which each tile is partitioned. In other words, the workload grows not only with the overall size of the acquired image, as it is intuitive, but also with the resolution of the microscope, since a larger map in terms of pixels has to be computed to correct alignment errors if the voxel size decreases.

Merging and multiresolution image generation (*fusion step* in the following) aim at creating a stitched image, i.e., a unique seamless image without overlapping regions in a form suitable for further processing. Indeed, one nice feature of TeraStitcher is that it enables the generation of multiple copies of the stitched image at decreasing resolution and size to simplify some types of manipulations when the highest resolution image is very large. Each low resolution image is obtained by properly combining nearby voxels and halving the size of the higher resolution image. We minimize memory occupancy and I/O operations that dominate this step by reading only once at the time limited portions of the input dataset and generating all the requested resolutions of that portion before loading another portion.

## 3. Parallelization Strategy

Both the alignment step and the fusion one can be applied to a portion of the image with no need to access other portions. Using command line options, the user can specify which portion has to be processed by TeraStitcher. By leveraging this feature, it is straightforward to implement a driver program that launches multiple instances working on different portions of the dataset. Every instance produces the corresponding output in parallel and when all instances have terminated, all outputs are properly *merged* so as to obtain the same final output that would be generated by a single instance of TeraStitcher when applied to the whole dataset.

Providing that the final merge step is computationally inexpensive, this approach has several advantages: (i) only the optimized sequential version of the code has to be maintained; (ii) no thread-safe code has to be introduced, possibly requiring variants in the shared data structures; (iii) the dataset partition strategy (i.e., the driver code) is kept separate from the internal algorithms of TeraStitcher; (iv) parallel stitching can be carried out also on a distributed memory platform (e.g., a cluster with a shared file system).

The driver program, referred to as *ParaStitcher* in the following, has been implemented in Python and uses MPI to execute in parallel multiple instances of TeraStitcher. Using the parameters of the MPI launcher, the user specifies the desired degree of parallelism *P* and the script, using the partition algorithms described in the following sections, divides the dataset into *N* partitions with *N* larger than *P* (e.g., ≥ 2*P*). After the script has performed some preliminary operations, it acts as a dispatcher, initially assigning *P* instances of TeraStitcher to as many MPI processes, and then assigning the remaining *N* − *P* instances on a FIFO basis to processes when they complete their assigned tasks. When all instances have been processed, the script performs the final merge operation and terminates. This way, a reasonable load balancing is attained in case dataset partitions do not generate the same workload.

ParaStitcher accepts exactly the same command line options of TeraStitcher making its use transparent for the user. The only exception is that it sets directly the command line options that control the portion of the dataset to be processed, in order to perform the correct partition of the tile matrix.

## 4. Alignment Step

### 4.1. Dataset Partition

The dataset before stitching can be viewed as a 2D matrix of regularly spaced tiles. As shown in Figure 1 each tile is a 3D matrix of voxels. TeraStitcher conventionally assumes that tiles are arranged as a matrix along dimensions X-Y and have all the same size along the three dimensions X, Y, and Z. Each tile has a nominal (i.e., imposed by the stages) fixed displacement with respect to the preceding tile in the matrix along the two dimensions. This displacement introduces some overlap between adjacent tiles (blue and red regions in Figure 1).

In the alignment step TeraStitcher reads a sub-block of the tile matrix specified through command line options, and computes all alignments among adjacent tiles in it. To make the use of the MIP-NCC algorithm more effective and to minimize memory occupancy and I/O operations, the two adjacent tiles are divided into sub-stacks and the algorithm is applied multiple times to homologous sub-stacks. Moreover, using suitable command line options, the alignment computation can be limited to a subset of consecutive sub-stacks. Of course, when applied to the whole dataset, TeraStitcher computes the alignments of all sub-stacks of all pairs of adjacent tiles in the tile matrix.

We highlight that, despite the significant I/O workload (obviously the whole dataset must be read at least once), the alignment step is CPU bound since the number of floating-point operations *per* data unit is high. As to the final merge operation that ParaStitcher has to perform, its cost is negligible, since it only consists into the processing of the xml files containing the alignments computed by each instance of TeraStitcher.

Turning our attention to the dataset partition algorithm, since the alignment algorithm is applied independently to homologous sub-stacks of adjacent tiles, if *S* is the number of sub-stacks in which each tile is divided, the easiest parallelization strategy is to launch *S* instances of TeraStitcher working on the whole tile matrix, but with each instance considering only intervals along Z corresponding to the sub-stack which is assigned to it. However, if *S* is not large enough, also the tile matrix should be partitioned into sub-blocks, with the *S* sub-stacks of each block assigned to an equal number of TeraStitcher instances.

**Figure 2:**
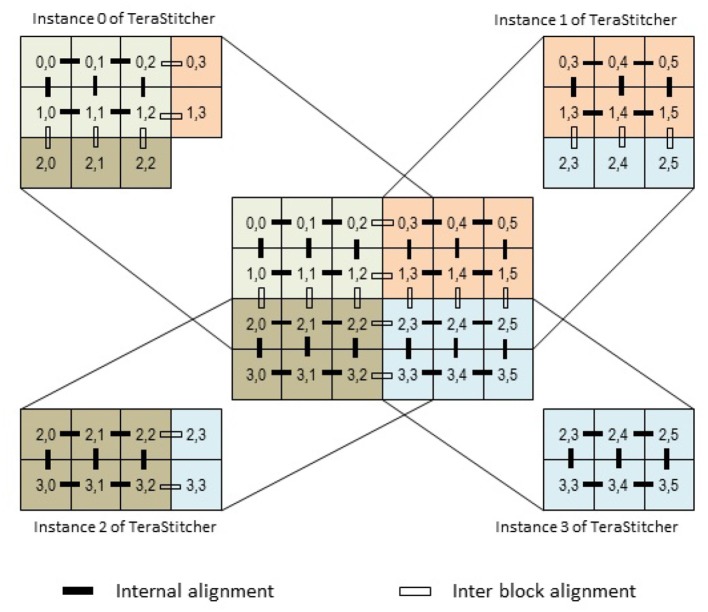
The tile matrix is partitioned in such a way that all alignments among adjacent tiles can be computed exactly once.

Hence, if *N* is the desired degree of the dataset partition, we can proceed as follows: if *S* ≥ *N* then split the dataset only along Z, otherwise split also the tile matrix into *B* = ⌈*N*/*S*⌉ sub-blocks. In the latter case, however, if we simply partition the tile matrix (i.e., the blocks of tiles are disjoint), the alignments between adjacent tiles that are on borders of different blocks (the *inter block* alignments of Figure 2) cannot be computed by any instance. To compute these alignments, tiles on the border of one block have to be loaded by both instances of TeraStitcher that have assigned the adjacent blocks, although just one of them should compute some alignments to avoid redundant computations.

To better understand the issue, consider a partition of the tile matrix, and let *b* be a generic block in the partition (see Figure 2). Let *i*, *j* be the indices of the last row and last column of *b*, respectively. The instance of TeraStitcher in charge of *b* must load and process also tiles of row *i* + 1 (unless *i* is the last row of the matrix) and tiles of column *j* + 1 (unless *j* is the last column in the matrix). However, for tiles in row *i*+1 only alignments against tiles in row *i* have to be computed, i.e., inter-blocks alignments, and similarly for tiles in columns *j* + 1 and *j*. Indeed, the other alignments against adjacent tiles are computed by other instances of TeraStitcher. The way tiles and alignment computations have to be assigned to different instances of TeraStitcher is depicted in Figure 2. Note that instance 0 of TeraStitcher does not need to load tile (2, 3) since all its alignments with respect to adjacent tiles are computed by other instances, and this is true for all partitions that are in charge of neither the last row nor the last column.

Based on these considerations, we added two command line options to TeraStitcher disabling the alignment computations among tiles belonging to the last row and to the last column of the block, respectively. This way, each instance of TeraStitcher is in charge of a block of the matrix partition which, according to its position in the tile matrix, is extended with one row at the bottom and one column at the right. TeraStitcher is then launched with the options disabling redundant alignment computations (see again Figure 2, where it is indicated which tiles are loaded by each instance of TeraStitcher and which alignments it computes).

We now discuss how the tile matrix can be optimally partitioned in, at least, *B* ≥ ⌈*N*/*S*⌉ blocks.

In general, it is not possible to estimate in advance the computational workload generated by a block of tiles since it depends, partially, on its contents. The safest policy is therefore to partition the matrix in blocks with approximately the same size, so that each instance of TeraStitcher must compute approximately the same number of alignments, and then rely on the load balancing effect induced when the number *N* of partitions is sufficiently greater than the number of computing units.

Second, as already explained, the partitioning of the tile matrix in blocks introduces some overhead. Although alignment computations are not duplicated, tiles on some block borders have to be loaded twice by instances of TeraStitcher [and a few of them even three times, see tile (2, 3) in Figure 2]. This entails additional I/O operations with respect to runs in which the tile matrix is not partitioned. It is easy to observe that, if the tile matrix has *m* rows and *n* columns, this overhead is measured by *m* times the number of vertical partitions minus one, plus *n* times the number of horizontal partitions minus one. For instance, with reference to Figure 2, the number of additional tile loads is 4 · 1 + 6 · 1 = 10.

As a consequence, an optimal partition must divide the tile matrix into, at least, *B* ≥ ⌈*N*/*S*⌉ blocks, minimizing the number of tiles that must be loaded multiple times.

To formalize the corresponding optimization problem, we model the tile matrix as a matrix of elements with *m* ≥ 2 rows and *n* ≥ 2 columns. Given a positive integer *B* ≤ ⌊*m*/2⌋ · ⌊*n*/2⌋ (the constraint is motivated by the need to avoid small partitions that would generate too much overhead), we want to partition the matrix in, at least, *B* blocks, minimizing a cost function under the constraint that: (i) the number of rows and columns of tiles in each block differ, at most, of 1, (ii) blocks have, at least, 2 rows and 2 columns.

A tile partition that fulfills the above conditions is identified by two integer numbers *p*_*m*_, *p*_*n*_ representing the partitions of rows and columns of the matrix, respectively, and satisfying the conditions:

$$1\leq p_{m}\leq m/2,\;1\leq p_{n}\leq n/2.$$

and:

(1) $$\begin{array}{r} p_{m}p_{n}\geq B. \end{array}$$

The cost function to be minimized, modeling the I/O overhead described above, is (*p*_*m*_ − 1)*n* + (*p*_*n*_ − 1)*m*. Our goal is therefore to solve the following optimization problem:

(2) $$\begin{array}{l} P(m,n,B)=\min\;(p_{m}-1)\;n+(p_{n}-1)\;m\\ \;1\leq p_{m}\leq m/2\\ \;1\leq p_{n}\leq n/2\\ \;p_{m}\cdot p_{n}\geq B\\ \;p_{m},p_{n}\text{ integers} \end{array}$$

which is not trivial, since it includes a non-linear constraint.

**Figure 3:**
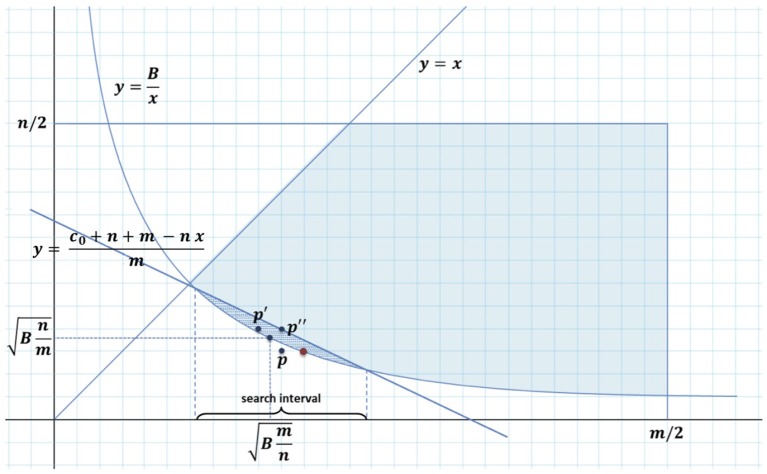
The solution of algorithm P(m,n,B) lies in the dashed area between hyperbola *y* = *B*/*x* and line *y* = (*c*_0_ + *nm* − *nx*)/*m*.

The problem can be represented on a Cartesian plane as in Figure 3, where the abscissa *x* represents the number of horizontal partitions and the ordinate *y* represents the number of vertical partitions.

We can easily find an approximate solution to P(m,n,B) if we consiter the three points:

$$\begin{array}{l} \;p=\left(\lceil \sqrt{Bm/n}\;\rceil ,\lfloor \sqrt{Bn/m}\rfloor \right)\\ p^{\prime }=\left(\lfloor \sqrt{Bm/n}\;\rfloor ,\lceil \sqrt{Bn/m}\rceil \right)\\ p^{\prime \prime }=\left(\lceil \sqrt{Bm/n}\;\rceil ,\lceil \sqrt{Bn/m}\rceil \right), \end{array}$$

that are the points with integer coordinates closest to the solution to P(m,n,B) when we relax the constraint that *p*_*m*_, *p*_*n*_ must be positive integers, and we choose the best of them satisfying (1) (*p*″ always does it).

A non trivial algorithm, starting from these three points, that it is guaranteed to find the optimal solution to P(m,n,B) is presented in the Appendix with a formal demonstration of its correctness. That algorithm has been implemented in the Python script to partition the dataset. However, it is worth noting that even using the best point among *p*, *p*′, and *p*″ provides a solution that coincides or it is very close to the optimum in all practical cases.

### 4.2. Multilevel Parallelism: The GPU Implementation

As already mentioned in section 2, the most time-consuming part of the alignment step is the evaluation of the NCC between the MIPs of overlapping regions. The NCC is a variant of the classic Cross Correlation in which data are normalized by subtracting the mean and dividing by the standard deviation of the two datasets (Lewis, 1995). Although cross correlation can be efficiently computed in the transform domain, its normalized version requires the manipulation of the original data, so that there is not a correspondingly simple and efficient transform domain expression. Moreover, in our case the so-called “feature” and “template” have the same size, so the NCC can be conveniently computed on a standard CPU using two simple double loops: the first double loop computes the averages of the two datasets. The second double loop computes the normalized correlation. Both loops could be parallelized by using, for instance, simple OpenMP directives that have the advantage of being portable between Windows and Unix systems. However, using multiple CPU cores to run in parallel those loops would interfere with the coarse grain parallelization strategy discussed in section 3, so we decided to pursue a different approach to speedup the evaluation of NCC, exploiting parallelism at a finer grain.

A possible solution is to use one or more Graphics Processing Units (GPUs) as floating point accelerators, since it is, by now, a common experience that GPUs can provide impressive speedups in data-intensive computations. One of the most successful software framework to program GPUs is CUDA (Nickolls et al., 2008), a software development kit (SDK) and application programming interface (API) that allows using simple extensions of the C programming language to code algorithms for execution on NVIDIA GPUs.

We implemented a CUDA version of NCC that carries out within a single *kernel* (the equivalent of a function invocation in CUDA) the entire computation for all the displacements of the pair of MIPs needed to build the NCC map mentioned in section 2. Each block of 1,024 *threads* in the CUDA grid executes one of the thousands of pairs of double-loops required for the evaluation of the NCC. More in detail, to compute the mean values of the two datasets, we leverage the so-called *shuffle* primitives that allow threads to exchange data using registers. Shuffle primitives may be used as building blocks of many reduction and scan operations^1^.

From the user viewpoint the computation of the NCC can switch from the CPU to GPU and viceversa by means of an environment variable. Also this version of TeraStitcher has been tested and runs under Linux, Mac OS and Windows.

## 5. Fusion Step

In the fusion step, given the indices of a 3D sub-matrix of the final stitched image, TeraStitcher reads the tiles in which the sub-matrix is contained, and generates (possibly many resolutions of) the corresponding region of the final image. When applied to the whole 3D matrix, TeraStitcher generates the complete stitched image.

With respect to parallelization, differently from the alignment step, the fusion step has the problem that data have to be written to secondary storage. This means that, in order to make parallelization effective, the format used to store the stitched image should support concurrent writes by multiple processes. Although this cannot guarantee actual parallelism in the I/O subsystem, since that depends on the features of the internal architecture, the point is that the format should not introduce unnecessary serialization of the accesses or, even worse, prevent from parallel writes.

Although most popular image formats do not support parallel writes, TeraStitcher supports a few file formats that allow concurrent writes of data belonging to different regions of the final image. For the sake of space, we briefly describe here two of these formats, which are representative of what can be done with others.

The simplest and currently most popular output format stores the image as a series of 2D images, each saved in a different file. Each 2D image usually corresponds to a plane in X-Y directions, whereas the series of files spans over the Z direction. Since all file systems support concurrent writes to different files, any dataset partition along Z can be used to parallelize the fusion step when this output format is used.

Storing the 3D image as a series of slices is simple, but it may be inefficient when the image is really big. Indeed, for very large images each slice may be as large as many Gbytes, which makes access to image sub-regions extremely slow if image files are not internally tiled. As an alternative to internal tiling, we proposed to use an external tiling, i.e., partitioning the image in multiple files, each storing a relatively small 3D sub-region of the 3D image (Bria and Iannello, 2012; Bria et al., 2016). This approach is simple and applicable independently of the specific format used to store individual tiles and it proved to be efficient in reducing the load time of small sub-regions of very large images (Bria et al., 2016). Moreover, it is also more effective for parallelization, since, differently from the series of 2D images, this form of tiling naturally supports any partition of the image into 3D sub-regions. For these reasons we will report in the following on results of experiments where the output format was of externally-tiled type.

Finally, we observe that the fusion step is clearly I/O bound, since the entire dataset must be read and written once (Bria and Iannello, 2012). The processing required for stitching is limited, unless compression is used (in reading, in writing or both) since in this case some processing must be done on all data. Hence, fusion step is I/O bound with possibly a non negligible fraction of processing.

As mentioned in Section 2, TeraStitcher can generate the output image at different resolutions. This feature coupled with the constraint that the tiles of the output image should be neither too large nor too small for efficiency reasons, makes non trivial the dataset decomposition for fusion step parallelization. Indeed, to avoid additional input operations, each instance of TeraStitcher must process a sub-region of the image large enough to enable the generation of all requested resolutions, with the additional constraint that, at all resolutions, tiles should have approximately the same size, not too smaller than a given value.

In order to formulate the partition problem, we preliminary observe that Terastitcher performs tiling independently over each dimension *i* on the basis of three parameters, all of which are integers:

- the image size *D*_*i*_ along dimension *i*, measured in voxels and assumed to be≫1;
- the ideal tile size *w*_*i*_ along dimension *i*, provided by the user and measured in voxels too;
- the lowest resolution to be generated that is determined by an integer *n*_*i*_ ≥ 0, 0 being the integer associated to the highest resolution (i.e., that of the acquired image).

For the sake of readiness, in the following we develop our discussion with respect to a single dimension and omit the index *i*. Indeed, all the results are immediately applicable independently to any dimension. Moreover, all quantities considered in the following are integers, unless differently specified. As to notations, we will use the symbol |*a*|_*b*_ to denote the modulo operation between the integers *a* and *b*.

Now, the constraints on dataset partition informally presented above imply that the tiling performed by a single instance of TeraStitcher when it processes the whole volume has the following properties: (i) the size of all tiles at all resolutions is no greater than *w* and not smaller than ⌈*w*/2⌉; (ii) all tiles have approximately the same size at all resolutions. Our goal is therefore to partition the interval of indices [0, *D* − 1] into sub-intervals and assign each sub-interval to a different instance of TeraStitcher in such a way that these two properties are satisfied at all resolutions as much as possible.

Since each instance must generate tiles from the sub-interval of voxels assigned to it, when halving is performed to generate lower resolutions, it is convenient, to minimize information loss, that sub-interval width be equal to $2^{n}\bar{b}$, with $\left\lceil w/2\right\rceil \leq \bar{b}\leq w$. This way, each instance generates 2^*n*^ tiles of size $\bar{b}$ at the highest resolution, 2^*n*−1^ tiles of the same size at the first lower resolution and so on, until it generates one tile, again of size $\bar{b}$, at the *n*-th lower resolution.

These can be easily done if there exists some $\bar{b}$ satisfying the above inequalities, and for which it is:

(3) $$\begin{array}{r} |D{|}_{2^{n}\bar{b}}=0. \end{array}$$

Indeed, in this case, *D* can be partitioned in $D/\left(2^{n}\bar{b}\right)$ sub-intervals and, at any resolution, tiles of size $\bar{b}$ can be generated.

If, conversely, (3) cannot be satisfied by any integer in the interval [⌈*w*/2⌉, *w*], the requested conditions can be reasonably satisfied by choosing $\bar{b}$, $\left\lceil w/2\right\rceil \leq \bar{b}\leq w$ in such a way that the interval [0, *D* − 1] is partitioned in $\left\lfloor D/\left(2^{n}\bar{b}\right)\right\rfloor$ sub-intervals of size $2^{n}\bar{b}$, plus one final sub-interval of maximum size $l^{\prime }=|D{|}_{2^{n}\bar{b}}$. This corresponds to solve the following optimization problem:

(4) $$\begin{array}{r} \bar{b}=\text{arg}\underset{\left\lceil w/2\right\rceil \leq b\leq w}{\max}|D{|}_{2^{n}b}. \end{array}$$

In this way the partition guarantees that:

- $\left\lceil w/2\right\rceil \leq \bar{b}\leq w$;
- at all requested resolutions, the tiles of the first $\left\lfloor D/\left(2^{n}\bar{b}\right)\right\rfloor$ sub-intervals are all equal to $\bar{b}$;
- the size of the last sub-interval is as close as possible to that of the others, implying that at any resolution the tile size is reasonably close to $\bar{b}$.

Hence, all tiles satisfy the conditions stated above, except those of the last sub-interval, if any, that are, however, reasonably guaranteed to have a size not too much smaller than the others. Moreover, it is worth noting that this way there is no loss of information at lower resolutions, except possibly a few voxels of the last sub-interval.

An algorithm that computes $\bar{b}$, *l*′, and the number *k* of sub-intervals in which [0, *D*−1] has been decomposed is presented in the Appendix, and it has been implemented in the Python script.

## 6. Results and Discussion

### 6.1. Alignment Step

In this Section we present the results of a set of numerical tests carried out to evaluate the performance achievable with the parallelization strategy above described.

The experiments have been carried out on an HP Z820 with 16 cores, 192 Gbytes RAM, one NVIDIA Tesla K20c, 9 TBs SATA disks in RAID 5 (6 TBs effective), and an IBM S822LC with 16 Power8 cores (SMT 8), 512 GByte RAM, 4 NVIDIA Tesla P100, 2 Solid State Disks (960 GByte each). The two systems represent different platforms to stress how our parallelization strategies behave ranging from a medium-range workstation to a high performance server. In particular, the two platforms have very different I/O subsystems whose throughput affects both the performance of read/write operations and the interaction with the GPUs.

We begin with the experimental results obtained on the HP workstation.

Since real datasets may differ greatly in size, and even datasets with similar size may be processed with different values of parameters to deal with different characteristics of the images, we have considered four different datasets and one of them (the largest one) has been processed with different parameters controlling the alignment algorithm. In Table 1 we report the characteristics of the datasets used in the experiments, the parameters used to control the alignment algorithm, the sequential execution time of the alignment step on the whole dataset in seconds, and the speedups achieved with 2, 4, and 8 processors. With more processors in all cases the attainable efficiency decreases because on the HP workstation the I/O fraction of the workload becomes the main bottleneck.

**Table 1:** Datasets characteristics, parameters of the alignment algorithm, sequential execution times, and speedups attainable with 2, 4, and 8 processors.

		**Size**	**Tile**	**Slice**		**Search**		**1**	**2**	**4**	**8**
**#**	**Dataset**	**(Gbyte)**	**matrix**	**size**	**#slices**	**area**	**Sub-stacks**	**proc (s)**	**procs**	**procs**	**procs**
1	Whole brain	223	3 × 3	2,048 × 2,048	2959	65 × 65	15	25680	1.94	3.69	6.58
2	Whole brain	223	3 × 3	2,048 × 2,048	2950	65 × 65	30	38880	1.89	3.48	6.00
3	Whole brain	223	3 × 3	2,048 × 2,048	2950	90 × 90	15	39060	1.88	3.54	6.45
4	Hippocampus	21	45 × 44	768 × 768	6	90 × 90	1	74820	1.96	3.75	7.03
5	Cerebellum	39	4 × 10	512 × 512	3701	60 × 60	19	14340	1.86	3.59	5.55
6	2-photon	2.65	6 × 9	1,568 × 1,568	10	58 × 33	1	524	1.87	3.20	5.63

The first three rows of the Table refer to the largest dataset we used and it will be referred in the following as “Whole brain”. It is actually a portion of a much bigger image of a whole mouse brain that was acquired at the European Laboratory for Non-Linear Spectroscopy (LENS) as a test image of their Confocal Light Sheet Microscope (Silvestri et al., 2012). Although its overall size is limited to 223 Gbytes, it is however representative of larger datasets that have similar tile size, and differ for the number of tiles only. Indeed, the speedups attainable on larger datasets in the same conditions (i.e., with the same controlling parameters) are quite similar to those reported in the first three rows of the table. For this dataset, three experiments have been carried out changing the search area and the number of sub-stacks per tile to be processed. In particular, experiments 2 and 3 require a very similar number of floating-point operations and almost twice the number of operations required by experiment 1. This is coherent with the measured sequential execution times, taking into account that in all cases the I/O workload is the same. In all cases the results confirm a pretty good scalability up to 8 processors. The other three experiments reported in the Table are representative of datasets with very different characteristics.

The dataset referred to as “Hippocampus” was acquired at LENS and was published in Allegra Mascaro et al. (2015). It has huge dimensions in X-Y (for a total of 1980 tiles) but very few slices. It tests the ability of the tile partition algorithm to generate an effective partition since no parallelism can be exploited along Z. The reported speedups show a very good performance confirming that the algorithm efficiently partitions the tile matrix keeping the I/O overhead at the bare minimum.

The dataset referred to as “Cerebellum” was acquired at LENS and was published in Silvestri et al. (2012). It has a smaller size in X-Y and a relatively small overall size. It represents a small dataset. Nevertheless, measured speedups are very similar to those of the “Whole brain” dataset, confirming that parallelization performance does not depend on the overall size of the dataset.

Finally, the dataset referred to as “2-photon” has been included because it is a very small dataset, again with many tiles and a few slices. It has been provided by users of TeraStitcher for testing purposes. Also in this case the parallelization strategy proves to be effective.

The results presented so far show that coarse grain parallelization leads to good speedups even on conventional cheap hardware. However, we carried out further experiments to evaluate how parallelization behaves when a high performance I/O subsystem is available, i.e., when the CPU bound nature of the problem is fully exposed. To that purpose we repeated experiment 1 on the second platform mentioned at the beginning of the section which has a sustained I/O throughput of almost 6 Gbytes/s. The results are shown in Table 2. Comparing speedups with those reported in the first row of Table 1, higher scalability is apparent, considering that an efficiency of 13.1/16 = 0.82 is achieved even with 16 processors.

**Table 2:** Performance of experiment #1 measured on the IBM server.

**Procs**.	**Wall time (s)**	**Speedup**
1	15300	1.00
2	8191	1.87
4	4100	3.73
8	2078	7.36
16	1168	13.10

We then tested the CUDA implementation. Results on the HP workstation are reported in Table 3. The advantage of using the GPU for computing the NCC is apparent. Moreover it is worth noting that coupling coarse and fine grain parallelization gives advantages in most cases and do not penalize the execution in the others, even though just one GPU is available. This is made possible by the overlap between processing and I/O that is naturally induced when the dataset is partitioned and more independent activities proceed concurrently.

**Table 3:** Speedups obtained using the GPU (Tesla K20c) available on the HP workstation.

**#**	**Dataset**	**1 proc. with CPU (s)**	**1 proc. with GPU**	**4 procs. with GPU**
1	Whole brain	25680	15.85	19.45
2	Whole brain	38880	24.00	23.14
3	Whole brain	39060	14.47	25.04
4	Hippocampus	74820	31.93	30.76
5	Cerebellum	14340	23.04	28.65
6	2-photon	524	23.82	27.58

*The completion times in seconds are reported from Table 1 for completeness*.

Finally, we tested the performance of the CUDA implementation on the IBM server. For the sake of brevity, we present the results just for experiment 1, because the others do not provide additional insights. Results are reported in Table 4. With one GPU the speedup is obviously greater than on the HP workstation, but the advantage is limited. Increasing the number of GPUs used and the degree of coarse grain parallelism exploited, performance notably increases, reaching an impressive speedup of more than two orders of magnitude when all available computing resources are used. We also ran the same experiment on a Titan-V, a latest generation NVIDIA card based on the Volta architecture. The host systems have different features (in particular the system hosting the Titan-V features has magnetic disks) so a comparison of the total elapsed time would not be fair. However, taking into account only the time required by the GPU to compute all NCCs, we pass from 70 to 42.5 s showing that the Volta architecture provides a clear advantage, probably due to its higher number of cores.

**Table 4:** Performance of experiment #1 using up to 4 GPUs on the IBM server.

**Procs**.	**#GPUs**	**Wall time (s)**	**Speedup (abs.)**	**Speedup (rel.)**
1	1	580	26.38	1.00
2	2	344	44.48	1.69
4	4	174	87.93	3.33
8	4	93	164.52	6.24
16	4	56	273.21	10.36

*Both absolute (i.e., wrt the sequential execution without the GPU) and relative (i.e., wrt the sequential execution with GPUs) speedups are reported*.

### 6.2. Fusion Step

Before presenting experimental data collected to assess the performance of ParaStitcher in the fusion step, we briefly discuss another issue that may cause overhead in the parallel working of TeraStitcher.

When the partition algorithm is applied to the Z dimension only, no overhead is introduced. Conversely, when the format of the output image is of an externally-tiled type and partition regards also X-Y dimensions, the instances of TeraStitcher have to read image data from all the tiles that have intersections with the partition they generate. This means that some tiles have to be loaded by different instances of TeraStitcher and therefore, differently from what happens in a completely sequential execution, those tiles are read more than once. That duplication of reading operations cannot be completely eliminated since tiles overlap, and therefore there is no way to partition the image in such a way that partitions correspond always to data coming from just one tile.

This source of overhead may further grow when input tiles have large size in X-Y. Indeed, it is convenient that tiles in the output image be not too large for efficient access in subsequent processing. Hence, if tiles of the unstitched input image are large, partitions are smaller than input tiles, and the number of TeraStitcher instances that need data from the same tile grows. This leads to duplicate reading operations because, although in principle only the data strictly needed should be read by each instance, in practice most image file formats (namely TIFF) do have limits in accessing image sub-regions, forcing instances to read also data that are read by other instances.

To evaluate the performance of the fusion step we used the same platform and datasets used for the performance evaluation of the alignment step. In Table 5 we report the experiments performed and the corresponding measured speedups. In all cases we generated the output at more than one resolution to evaluate how the partition Algorithm 2 works. Although dataset characteristics are the same reported in Table 1, for the sake of readability we have duplicated columns corresponding to parameters relevant to evaluate the overhead. For the same reason, in the table are reported the percentage of overlap between adjacent tiles and the tile size of the output image. As already stated, all experiments generate an externally tiled image.

**Table 5:** Datasets characteristics, parameters of the fusion algorithm, sequential execution times, and speedups attainable with 2, 4, 6, 8, 10, and 12 processors.

				**Overlap**	**Output**	**1**	**2**	**4**	**6**	**8**	**10**	**12**
**#**	**Dataset**	**Slice size**	**#slices**	**(%)**	**tile size**	**proc (s)**	**procs**	**procs**	**procs**	**procs**	**procs**	**procs**
1	Whole brain	2048 × 2048	2959	25	768 × 768 × 256	7470	1.81	2.25	2.29	2.05	n.a.	n.a.
2	Cerebellum	512 × 512	3701	27	384 × 384 × 384	1886	1.81	3.18	4.76	5.10	6.83	7.23
3	Hippocampus	768 × 768	6	24	512 × 512 × 6	760	1.82	3.29	4.22	4.47	5.20	6.08
4	2-photon	1568 × 1568	10	7/4	768 × 768 × 10	75	1.92	3.13	4.17	5.00	6.25	5.36

*In the “2-photon” dataset the percentage of overlap is different along X and Y dimensions*.

The four datasets have different characteristics that allow us to evaluate parallelization of fusion step in different conditions. More specifically we have that:

- the “Whole brain” dataset has many slices, a large tile size in X-Y, and a fairly large overlap, especially in absolute terms;
- the “Cerebellum” dataset has many slices too, but with fairly small tile size in X-Y, and a fairly large overlap in percentage, but relatively small in absolute terms;
- the “Hippocampus” dataset has very few slices, a fairly small tile size in X-Y, and a fairly large overlap in percentage, but again relatively small in absolute terms;
- the “2-photon” dataset has very few slices and a large tile size in X-Y, but with a very limited overlap.

Taking into account these characteristics, the previous considerations about the input overhead, and the highly I/O bound nature of the fusion step, reported speedups suggest that:

- The partition algorithm works well since in all cases, except the “Whole brain” dataset, speedups are quite good up to 6 processors and in one case up to 10 processors;
- The “Whole brain” dataset suffers of both the large tile size and the large absolute overlap; indeed for 2 processors the partition algorithm applies to Z dimension only and the speedup is comparable with that of other datasets because there is no overhead, but already for 4 processors, even dimensions X-Y must be partitioned to guarantee some load balancing; this causes a rapid growth of input overhead so that parallelization becomes inefficient for 6 processors and completely useless for more processors.

### 6.3. Comparison With BigStitcher

As introduced in section 2, the only noncommercial tool that supports 3D stitching of Terabyte-sized images and uses parallelism to boost performance is BigStitcher (Hörl et al., 2018). Similarly to TeraStitcher, BigStitcher divides the stitching pipeline in five steps: dataset import, pairwise displacement computation, filtering pairwise displacements, globally optimal tile placement, and image fusion generation. This made possible to directly compare the performance of the two tools on the two most time consuming steps, namely the alignment and the fusion step. For this comparison, we used the HP workstation and the four datasets described in section 6.1. We executed all experiments using always the default parameters and algorithms, except when a different choice was suggested by the characteristics of the dataset at hand (e.g., the tiles overlap), or by the BigStitcher documentation (e.g., the caching type in the fusion step). Even though the degree of parallelism used by BigStitcher was not under user control, we verified that all processor cores were fully used during the execution in all experiments.

Results of the experiments for the alignment step are reported in Table 6. BigStitcher was 1.52 − 2.45× faster than ParaStitcher when GPUs were not used, whereas ParaStitcher was 2.00 − 3.39× faster than BigStitcher when GPUs were used. For a better evaluation of these results, it is worth noting that BigStitcher, besides thread-level parallelism, uses by default a (4,4,2) downsampling in X,Y,Z dimensions to speedup pairwise displacement computation and then uses an *n*-dimensional implementation of a quadratic fit to achieve subpixel accuracy.

**Table 6:** Comparison in pairwise displacement computation performance between ParaStitcher and BigStitcher.

	**ParaStitcher**		**BigStitcher**
**Dataset**	**CPU** **(8 procs.)**	**CPU + GPU** **(4 procs.)**	**(48 threads + downsampling)**
Whole brain	3903	1320	n.a.
Hippocampus	10643	2432	n.a.
Cerebellum	2584	501	1699
2-photon	93	19	38

*All times are measured on the HP workstation and are in seconds*.

Unfortunately, despite multiple attempts, BigStitcher was not able to complete the alignment step for the “Whole brain” and the “Hippocampus” datasets. Specifically, after it started and completed a number of pairwise computations, BigStitcher hanged and freezed all Fiji windows for hours. When BigStitcher was launched on a smaller subset (8 × 44 tiles) of the “Hippocampus” dataset (45 × 44 tiles), the computation terminated successfully. However, it hanged again when launched on a smaller subset (2 × 2 tiles) of the “Whole brain” dataset (3 × 3 tiles). We also observed that BigStitcher used a considerable amount of RAM, up to 1.5× the dimensions of the input dataset. Although it is not possible to establish a direct connection between these issues in the absence of requirements specifications and without controlling the degree of parallelism, these behaviors suggest that the current release of BigStitcher (which is still in a Beta-version) can have problems when the size of the dataset grows, possibly depending on the structure (number of tiles and/or slices, file format, etc.) of the source dataset.

As to the fusion step, BigStitcher can generate only a single 16/32 bits 3D image^2^ in either OME-TIFF or HDF5 BigDataViewer format. When the fused image is generated in the former format, parallelization cannot be exploited in writing operations, whereas this would be possible in principle when the latter format is used, since a thread-safe version of the HDF5 library exists. Nevertheless, we observed that fusion timings of BigStitcher remarkably grow when the BigDataViewer format is chosen. While this observation can be explained by the characteristics of the format (high compression ratio and more complex internal structure than TIFF), it suggests that parallelism is not exploited or it is not effective in the current release of BigStitcher. Conversely, ParaStitcher can generate formats where the fused image is split in many files (a series of 2D slices or a 3D grid of tiles). These formats allow an effective parallelization of the image fusion step as demostrated by the experiments discussed above and by data reported in Table 7.

**Table 7:** Comparison in image fusion performance between ParaStitcher and BigStitcher.

	**ParaStitcher**			**BigStitcher**
**Dataset**	**Single 3D TIFF**	**2D series**	**3D grid of tiles**	**Single 3D TIFF**
	**(Sequential)**	**(Parallelized)**	**(Parallelized)**	**(48 threads)**
Cerebellum	3814	242	261	3576
2-photon	28	10	10	425

*All formats, except the single 3D TIFF, are compressed and include multiple resolutions*.

*All times are measured on the HP workstation and are in seconds*.

From this brief analysis we can conclude that BigStitcher definitely exploits parallelism to speedup the pairwise displacement computation, although it is difficult to give a precise performance characterization since the degree of parallelism cannot be varied. Data reported in Table 6, however, confirm that ParaStitcher can exploit both coarse- and fine-grained parallelism to achieve a dramatic reduction of processing times even on the two datasets where BigStitcher failed. As to image fusion, ParaStitcher turns out to be the only tool capable to effectively exploit parallelism in this step.

The interested reader may find a comparison between the two tools with respect to stitching parallelization in Table 8, where we summarize all the abovereported findings and point out some other key differences^3^.

**Table 8:** Comparison between ParaStitcher and BigStitcher (Beta-version) on stitching parallelization.

	**ParaStitcher**	**BigStitcher**
Input arrangement	Regular grid of tiles (row-by-row), sparse regular grid by XML specification	Regular grid of tiles (various arrangements), non-regular grid
Input format	TIFF (multipage or 2D series), Bitplane Imaris, Hamamatsu DCIMG, OpenCV formats	TIFF (multipage only), Bioformats, Zeiss Lightsheet Z.1, MicroManager diSPIM
Multi-channel	Yes	Yes
Alignment parallelization	CPU (multi-process) + GPU (CUDA) user-controllable 2.00 − 3.39× faster than BigStitcher ^*a*^	CPU (multi-thread) cannot be controlled by user
Fusion parallelization	CPU (multi-process) user-controllable 4.25 − 14.78× faster than BigStitcher ^*a*^	no
Memory usage	< 0.02 × #procs× the size of input dataset	≈1.5× the size of input dataset ^*a*^
Output format ^*b*^	Compressed/uncompressed 8/16 bits 2D TIFF series, Compressed/uncompressed 8/16 bits multipage TIFF	n.a.
Output arrangement ^*b*^	Regular grid of tiles (row-by-row), Series of whole 2D slices	n.a.

a *measured on the “Cerebellum” and “2-photon” datasets, whereas on the other two datasets “Whole brain” and “Hippocampus” BigStitcher did not complete the alignment step*.

b *we reported only the formats/arrangements that can be effectively generated in parallel by the tools, i.e., sequentially written formats/arrangements have been omitted here*.

## 7. Conclusions and Future Works

We have presented an approach aiming at reducing the stitching time of TeraStitcher using parallel processing at different grain levels. At coarse level, parallelization is achieved exploiting the possibility of processing concurrently different partitions of the image, and designing two efficient algorithms to perform the dataset partition. At fine level, data parallelism characterizing the most computing intensive part of the alignment algorithm is exploited using GPUs. Both levels of parallelism can be coupled providing significant speedups on a common medium range workstation and close to three hundreds fold speedup on a high end server. Additional advantages of the proposed approach are that parallelization does not require additional efforts for the maintenance of TeraStitcher code, no special image file formats are introduced, there are no changes in the user interface, apart the need to specify the desired level of parallelism, and execution on distributed memory platforms is possible.

Several directions are worth exploring to further improve the user experience in stitching vary large images. For example, the alignment step could be further accelerated by computing the NCC map on a downsampled image followed by a refinement in the original resolution. This approach should be adapted to preserve the effectiveness of the reliability estimator used by TeraStitcher to filter out unreliable computed displacements. Also, the dramatic improvement in alignment computation provided by GPUs makes the I/O issues of the fusion step the real bottleneck in the stitching pipeline. Two different approaches are possible to overcome this limitation: (i) to reduce the I/O workload (e.g., by using lossy compression compatible with application requirements) combined with file formats that can better exploit high performance I/O subsystems; (ii) to skip the fusion step and process directly the original dataset through the displacement data provided by the alignment step. As a matter of fact, we are currently working in this last direction, that we refer to as “stitching-on-the fly,” by extending Vaa3D-Terafly (Bria et al., 2015; Bria et al., 2016) to support visualization and annotation of very big images without the need to perform the fusion step.

## Data Availability

Datasets “Whole brain,” “Hippocampus,” and “Cerebellum” are not publicy available due to their size and because they have been conceded to us for testing only by colleagues from LENS. Nevertheless, we may ask permission to distribute them to interested people on reasonable request. The only expception is the “2-photon” dataset which is available from the TeraStitcher project's site.

## Author Contributions

GI and AB conceived the study and implemented the sequential part of TeraStitcher. GI designed the algorithms for dataset partitioning. MG and GI implemented the Python scripts driving the parallelization. MB implemented the CUDA code of the MIP-NCC algorithm. GI and MB carried out the performance measurements. GI wrote the paper with input from all authors.

### Conflict of Interest Statement

The authors declare that the research was conducted in the absence of any commercial or financial relationships that could be construed as a potential conflict of interest.

## Footnotes

^1^For details see at https://devblogs.nvidia.com/cuda-pro-tip-kepler-shuffle

^2^For the sake of comparison, we are not considering here time-lapse and/or multi-view datasets.

^3^The comparison focuses only on stitching. It is worth noting, however, that BigStitcher provides several other functionalities such as multi-view reconstruction, deconvolution, and illumination selection.

## Abbreviations

LENS: European Laboratory for Non-linear Spectroscopy
MIP: Maximum Intensity Projection
NCC: Normalized Cross- Correlation
TB: TeraByte.
