# Supplementary material for: Exploiting Multi-Level Parallelism for Stitching Very Large Microscopy Images
Source: Front Neuroinform. 2019 Jun 4;13:41. doi: 10.3389/fninf.2019.00041 (PMC6558144; doi:10.3389/fninf.2019.00041)
Supplement: Supplementary file 1 [file Data_Sheet_1.pdf]

Appendix[1]

Optimal solution to $\mathcal{P}(m, n, B)$

The problem $\mathcal{P}(m, n, B)$ is optimally solved by Algorithm 1.

To demonstrate its correctness we preliminarily observe that the assumptions:

$$m \geq 2, \quad n \geq 2, \quad B \leq \lfloor m/2 \rfloor \cdot \lfloor n/2 \rfloor, \tag{5}$$

guarantee that $\mathcal{P}(m, n, B)$ has definitely a solution. Moreover, in the following, we assume, without loss of generality, that $m \geq n$, unless otherwise specified. The following lemma holds.

Lemma 1 *If p_m, p_n are solutions of $\mathcal{P}(m, n, B)$, then $p_m \geq p_n$.*

Proof. For $m = n$, if $p_m < p_n$ we can swap the two values without changing the cost of the solution which remains optimum. For $m > n$, we prove the lemma by contradiction. Let us assume $p_m < p_n$. Since $m > n$, we have that $(p_n - 1)n + (p_m - 1)m < (p_m - 1)n + (p_n - 1)m$, but this is impossible because p_m, p_n are solutions of the problem. □

Lemma 1, and conditions (1) and (5) lead us to the conclusion that the solution to $\mathcal{P}(m, n, B)$ must be a point with integer coordinates in the shaded area shown in Figure 3, including its border. Moreover, lines of equation:

$$y = \frac{c + n + m - n\,x}{m}.$$

are the locus of points that have cost c.

To solve problem $\mathcal{P}(m, n, B)$ we can initially relax the constraint that p_m, p_n must be positive integers. In this case the minimum cost is:

$$\hat{c} = \sqrt{B\,\frac{m}{n}}\,n + \sqrt{B\,\frac{n}{m}}\,m - (n + m). \tag{6}$$

The solution of $\mathcal{P}(m, n, B)$ must be a point $(\bar{p}_m, \bar{p}_n)$, with integer coordinates, above or lying on the hyperbola (see Figure 3):

$$y = \frac{B}{x}. \tag{7}$$

[1]Some figures and equations numbers refer to those reported in the paper.

```
ALGORITHM (p_m, p_n, c) = P(m, n, B) :            % PRE: m ≥ n
  p_m := ⌈ √(B m/n) ⌉; p_n := ⌊ √(B n/m) ⌋
  if p_m p_n ≤ B then                             % p does not satisfy (1)
    p_m := ⌊ √(B m/n) ⌋; p_n := ⌈ √(B n/m) ⌉
    if p_m p_n ≤ B then                           % p' does no satisfy (1)
      p_m := ⌈ √(B m/n) ⌉                         % p'' definitely satisfy (1)
    end
  end
  c := m p_n + n p_m                              % cost of (p_m, p_n)
  p_{m_0} := p_m                                  % save p_{m_0}
  % look for a better solution by increasing p_m
  p_{m_cur} := p_{m_0} + 1; p_{n_cur} := (c − n p_{m_cur})/m
  while p_{m_cur} ≤ m/2 ∧ p_{m_cur} p_{n_cur} ≥ B do
    if 1 ≤ ⌊ p_{n_cur} ⌋ ∧ p_{m_cur} ⌊ p_{n_cur} ⌋ ≥ B then
      % update current candidate and its associated cost
      p_m := p_{m_cur}; p_n := ⌊ p_{n_cur} ⌋
      c := m p_n + n p_m
    end
    % look for an even better solution, if any
    p_{m_cur} := p_{m_cur} + 1
    p_{n_cur} := (c − n p_{m_cur})/m
  end
  % look for a better solution by decreasing p_m starting from p_{m_0}
  p_{m_cur} := p_{m_0} − 1; p_{n_cur} := (c − n p_{m_cur})/m
  while ⌊ p_{n_cur} ⌋ ≤ p_{m_cur} ∧ p_{m_cur} p_{n_cur} ≥ B do
    if ⌊ p_{n_cur} ⌋ ≤ n/2 ∧ p_{m_cur} ⌊ p_{n_cur} ⌋ ≥ B then
      % update current candidate and its associated cost
      p_m := p_{m_cur}; p_n := ⌊ p_{n_cur} ⌋
      c := m p_n + n p_m
    end
    % look for an even better solution, if any
    p_{m_cur} := p_{m_cur} − 1
    p_{n_cur} := (c − n p_{m_cur})/m
  end
```

Algorithm 1: Pseudo-code of the algorithm solving $\mathcal{P}(m, n, B)$.

and as near as possible to line:

$$y = \frac{\hat{c} + n + m - n\,x}{m}. \tag{8}$$

Consider now the points (see Figure 3):

$$\begin{aligned} p &= \left(\left\lceil \sqrt{B\,m/n} \right\rceil, \left\lfloor \sqrt{B\,n/m} \right\rfloor\right) \\ p' &= \left(\left\lfloor \sqrt{B\,m/n} \right\rfloor, \left\lceil \sqrt{B\,n/m} \right\rceil\right) \\ p'' &= \left(\left\lceil \sqrt{B\,m/n} \right\rceil, \left\lceil \sqrt{B\,n/m} \right\rceil\right) \end{aligned}$$

Remembering that $m \geq n$, there is a non decreasing cost associated to p, p', and p''. Moreover, p'' definitely satisfies conditions (1), as it can be easily verified using assumptions (5).

Based on these considerations, we consider the point:

$$(p_{m_0}, p_{n_0}) = \begin{cases} \left(\left\lceil \sqrt{B\,m/n} \right\rceil, \left\lfloor \sqrt{B\,n/m} \right\rfloor\right) & \text{if } p \text{ satisfies conditions (1)} \\ \left(\left\lfloor \sqrt{B\,m/n} \right\rfloor, \left\lceil \sqrt{B\,n/m} \right\rceil\right) & \text{if } p \text{ does not satisfy conditions (1) and } p' \text{ does} \\ \left(\left\lceil \sqrt{B\,m/n} \right\rceil, \left\lceil \sqrt{B\,n/m} \right\rceil\right) & \text{otherwise.} \end{cases}$$

This point is the best candidate solution to $\mathcal{P}(m, n, B)$ among these three points, and it can be easily verified that $(p_{m_0} \geq p_{n_0})$, as requested by Lemma 1. The cost associated to (p_{m_0}, p_{n_0}) is:

$$c_0 = (p_{m_0} - 1)\,n + (p_{n_0} - 1)\,m.$$

The solution of $\mathcal{P}(m, n, B)$ must therefore have a cost belonging to the interval $[\hat{c}, c_0]$, and it must be a point in the area A delimited by the hyperbola (7) and the line:

$$y = \frac{c_0 + n + m - n\,x}{m}, \tag{9}$$

including the border (the dashed area in Figure 3).

We can now prove the following lemma.

Lemma 2 *For any given integer h, there is at most one integer k such that point (h,k) is a candidate solution of $\mathcal{P}(m,n,B)$, i.e. (h,k) is in A.*

Proof. Let us consider first the case when p satisfies conditions (1). For any given abscissa x, it is:

$$\frac{c_0 + n + m - n\,x}{m} - \frac{\hat{c} + n + m - n\,x}{m} = \frac{c_0 - \hat{c}}{m} < 1.$$

because we have:

$$\begin{aligned} \frac{c_0 - \hat{c}}{m} &= -\left(\sqrt{B\,n/m} - \left\lfloor \sqrt{B\,n/m} \right\rfloor\right) + \frac{n\left(\left\lceil \sqrt{B\,m/n} \right\rceil - \sqrt{B\,m/n}\right)}{m} \\ &= -\alpha + \frac{n\,\beta}{m} < 1, \end{aligned}$$

since $0 \le \alpha, \beta < 1$ and $m \ge n$. Hence, there can be at most one point with integer coordinates between lines (8) and (9). The thesis follows from the observation that the area of interest is between the two lines.

The case when p does not satisfy conditions (1), but p' does, can be proved in a similar way.

If conversely, neither p nor p' satisfy conditions (1), for $h = \left\lceil \sqrt{B\,m/n} \right\rceil$, the difference between the ordinates of hyperbola (7) and line (9) is less than 1, since p'' lies on (9), p is below (7), and the difference of their ordinates is less than 1. The same holds for $h = \left\lfloor \sqrt{B\,m/n} \right\rfloor$ because the point on line (9) has ordinate:

$$\frac{c_0 - n\left\lfloor \sqrt{B\,m/n} \right\rfloor}{m} = \left\lceil \sqrt{B\,n/m} \right\rceil + \frac{n\left(\left\lceil \sqrt{B\,m/n} \right\rceil - \left\lfloor \sqrt{B\,m/n} \right\rfloor\right)}{m},$$

the difference with the ordinate of p' is:

$$\frac{n\left(\left\lceil \sqrt{B\,m/n} \right\rceil - \left\lfloor \sqrt{B\,m/n} \right\rfloor\right)}{m} \le 1,$$

and p' is below hyperbola (7).

Now, for $h < \left\lfloor \sqrt{B\,m/n} \right\rfloor$ and $h > \left\lceil \sqrt{B\,m/n} \right\rceil$, the difference of ordinates of hyperbola (7) and line (9) further decreases, so again at most one point with integer ordinate can be in the area of interest. □

The search of the optimal solution can now proceed by considering the point:

$$(p_{m_1}, p'_{n_1}) = \left(p_{m_0} + 1, \frac{c_0 + n + m - n\,(p_{m_0} + 1)}{m}\right),$$

lying on line (9). If this point is below the hyperbola (7), then we are outside the region of interest and it is pointless to further increment the x coordinate along line (9). If this is not the case, let us consider the point $(p_{m_1}, p_{n_1}) = \left(p_{m_0} + 1, \left\lfloor \frac{c_0+n+m-n\,(p_{m_0}+1)}{m} \right\rfloor\right)$. If it satisfies (1), then it is a better candidate solution, since its associated cost:

$$c_1 = p_{m_1}\, n + p_{n_1}\, m - (n + m),$$

is less than or equal to c_0, since (p_{m_1}, p_{n_1}) is on line (9) or below it. Hence, the line:

$$y = \frac{c_1 + n + m - n\,x}{m}, \tag{10}$$

can substitute line (9) in the search.

It is worth noting that no point below (p_{m_1}, p_{n_1}) can be a solution of $\mathcal{P}(m, n, B)$, because of Lemma 2. The search can therefore continue the same way by increasing p_{m_1} and checking if the next point lying on the line passing for the last candidate solution is above hyperbola (7).

When, moving on the current cost line a point below hyperbola (7) is reached, the search for increasing p_m terminates, and a similar search must be carried on for decreasing p_m, starting from $p_{m_0} - 1$. This search terminates when either a point below hyperbola (7) is reached, or the first coordinate of the next point is less than the second one (as requested by Lemma 1).

The last considered candidate solution satisfying (1) is the solution $(\bar{p}_m, \bar{p}_n)$ of $\mathcal{P}(m, n, B)$.

To the purpose of evaluating the complexity of the algorithm, let us consider the two intersections between hyperbola (7) and line (9), and let I be the *search interval* between their projections on the horizontal axis (see again Figure 3).

The algorithm considers only points on (9) with integer abscissa in I, and for each of them executes a constant number of steps. Indeed, not all integers in I are actually considered because of the conditions $1 \leq p_m \leq m/2$, $1 \leq p_n \leq n/2$, and $m \geq n$. Moreover, any time a better solution is found, a new line corresponding to a lower cost substitutes (9), which reduces the width of the search interval. Hence, the number of

integers in I, i.e. its width, is an upper bound for the algorithm complexity.

The lower and upper bounds of I are the solutions of the equation:

$$n\,x^2 - c_0'\,x + m\,B = 0,$$

where $c_0' = c_0 + n + m$ to simplify notation. Hence the interval width is:

$$\frac{\sqrt{(c_0'^2 - 4\,m\,n\,B)}}{n} = \sqrt{(c_0'/n)^2 - 4\,m\,B/n)}. \tag{11}$$

Now, c_0' has the form $m\,K_1 + n\,K_2$, where $K_1 \le \left\lceil \sqrt{B\,n/m} \right\rceil < \sqrt{B\,n/m} + 1$ and $K_2 \le \left\lceil \sqrt{B\,m/n} \right\rceil < \sqrt{B\,m/n} + 1$. Hence, substituting these bounds in (11) and carrying out some calculations, the width of I is definitely less than:

$$\sqrt{\left(\frac{m}{n} + 1\right)^2 + 4\left(\frac{m}{n} + 1\right)\sqrt{B\,\frac{m}{n}}}.$$

which is $O(B^{1/4})$ if we assume that m/n is upper bound, as it is reasonable since values of m/n much larger than 1 are unlikely.

In practice, however, the algorithm requires a number of steps bound by a small constant (running some simulations we found an upper bound of 6), since either (p_{m_0}, p_{n_0}) is already a very good solution inducing a very small I, or better solutions are found in the first steps of the algorithm, which greatly reduces the search space leading to a quick termination.

Algorithm for partitioning the dataset in the fusion step

With reference to the notation introduced in Section 5, Algorithm 2 computes $\bar{b}$, l', and the number k of sub-intervals in which $[0, D-1]$ has been decomposed.

To explain how the algorithm works, let be $b_h = w - h$ with $h = 0, 1, \ldots, \lfloor w/2 \rfloor$ and $k_h = \left\lfloor \frac{D}{2^n b_h} \right\rfloor$. For some $h \le \lfloor w/2 \rfloor$, let be:

$$r_h = D - k_h 2^n b_h < 2^n b_h. \tag{12}$$

If $r_h = 0$, b_h is an ideal solution which induces a partition over $[0, D-1]$ of k_h sub-intervals, all of size $2^n b_h$.

Conversely, when $r_h > 0$, b_h induces a partition of $[0, D-1]$ into k_h sub-intervals of

```
ALGORITHM (b̄, l′, k) = F(D, w, n) :
  b̄ := w; l′ := |D|_{2^n w}; k = ⌊D/(2^n w)⌋;
  h := 0;
  while true do
     b_h := w − h;
     k_h = ⌊D/(2^n b_h)⌋;
     if |D|_{2^n b_h} = 0 then                 % this is the largest ideal solution
        b̄ := b_h; l′ := 0; k = k_h;           % update results
        break                                   % exit
     elseif |D|_{2^n b_h} > r then             % this is a better solution
        b̄ := b_h; l′ := |D|_{2^n b_h}; k = k_h; % update results
     end
     if h = ⌊w/2⌋ then                         % this is the best possible solution
        break                                   % exit
     end
     h := min(⌊w/2⌋, h + max(1, ⌊b_h − D/(2^n(k_h+1))⌋));
  end
  if |D|_{2^n b_h} > 0 then                    % there is one more sub-interval
     k = k + 1;
  end
```

Algorithm 2: Pseudo-code of the algorithm for finding sub-intervals of $[0, D-1]$.

size $2^n b_h$, plus one additional sub-interval of size $r_h < 2^n b_h$. We then consider if we can replace b_h with $b_{h+\alpha}$, $\alpha > 0$ and integer such that it is:

$$r_{h+\alpha} = D - k_h 2^n b_{h+\alpha} \leq 2^n b_{h+\alpha}, \tag{13}$$

that is, if we can find a value $b_{h+\alpha} < b_h$ that still induces a partition of $[0, D-1]$ into k_h sub-intervals of size $2^n b_{h+\alpha}$ plus one additional sub-interval of size $r_{h+\alpha} \leq 2^n b_{h+\alpha}$. It is worth noting that if $b_{h+\alpha}$ exists, it is a better choice than b_h since $r_{h+\alpha} > r_h$. In particular, we are interested in finding the largest value of α that satisfies (13), since this is the best value that induces a partition of $[0, D-1]$ into $k_h + 1$ sub-intervals.

Since (13) can be rewritten as:

$$D - k_h 2^n b_{h+\alpha} = D - k_h 2^n (b_h - \alpha) \leq 2^n b_{h+\alpha} = 2^n (b_h - \alpha), \tag{14}$$

the largest α we are looking for is:

$$\alpha = \left\lfloor \frac{2^n b_h (k_h + 1) - D}{2^n (k_h + 1)} \right\rfloor = \left\lfloor b_h - \frac{D}{2^n (k_h + 1)} \right\rfloor, \tag{15}$$

and $b_{h+\alpha}$ is:

$$b_{h+\alpha} = b_h - \alpha \;\; = \;\; b_h - \left\lfloor b_h - \frac{D}{2^n(k_h+1)} \right\rfloor = \left\lceil \frac{D}{2^n(k_h+1)} \right\rceil .$$

Note that a $b_{h+\alpha} < b_h$ could not exist, i.e., there is no integer $\alpha > 0$ satisfying (14). Indeed, even though $r_h > 0 \implies b_h > \frac{D}{2^n(k_h+1)}$ from (12), the difference between the two sides of the inequality could be less than 1, meaning that (15) is 0. In this case, b_h is the best value that induces a partition of $[0, D-1]$ into $k_h + 1$ sub-intervals. However, increasing h could still generate a better solution with more sub-intervals, hence, as long as h is smaller than $\lfloor w/2 \rfloor$, h should be incremented and the procedure repeated.

To evaluate complexity of algorithm 2, we observe that, at step 15, h increases at least by 1, and that step 12 limits its growth up to $\lfloor w/2 \rfloor$. This implies that the algorithm terminates at most after $\lfloor w/2 \rfloor$ cycles. Nevertheless, a possibly better bound can be derived by considering that the value of hidden variable k_h increases at least every 2 cycles of the algorithm. Indeed, if some execution of step 15 simply skips non optimal values of b_h, and therefore k_h does not actually change at step 5 , it definitely increases at the next cycle, since h cannot be increased further keeping the the same number of partitions. Now, k_h can vary from the initial value $\left\lfloor \frac{D}{2^n w} \right\rfloor = k_0$ to its maximum possible value $\left\lfloor \frac{D}{2^n \lceil w/2 \rceil} \right\rfloor$. Hence the algorithm can execute at most $2\left(\left\lfloor \frac{D}{2^n \lceil w/2 \rceil} \right\rfloor - \left\lfloor \frac{D}{2^n w} \right\rfloor \right) < 2(k_0 + 1)$ cycles, and $2(k_0 + 1) < \lfloor w/2 \rfloor$ in practical cases.
